# Editorial: Environmental determinants of athletes’ development and performance

**DOI:** 10.3389/fspor.2026.1840848

**Published:** 2026-04-21

**Authors:** Mabliny Thuany, Pedro Forte, Thayse Natacha Gomes

**Affiliations:** 1 Sports Department, State University of Pará, Belém, Brazil; 2 Ultra Sports Science Foundation, Pierre, France; 3 Department of Sports, Higher Institute of Educational Sciences of the Douro, Penafiel, Portugal; 4 Department of Sports Sciences, Instituto Politécnico de Bragança, Bragança, Portugal; 5 Research Center for Active Living and Wellbeing (Livewell), Instituto Politécnico de Bragança, Bragança, Portugal; 6 Department of Physical Education, Federal University of Sergipe, São Cristóvão, Brazil

**Keywords:** athletes, development, environment, performance, sport

Talent development and sports performance are shaped by a plethora of factors, including both individual and environmental determinants. While the role of individual attributes in athletes’ development and performance has been highlighted in the scientific literature, there is a growing interest in understanding the role played by environmental factors (2), in different outcomes and across different levels and contexts. The Research Topic “Environmental Determinants of Athletes’ Development and Performance” aimed to investigate the relationship between the environment and sports, from athlete development through to peak performance.

The nine studies included in this research topic reinforce the multifactorial nature of athlete development by highlighting the dynamic interaction between psychological, social, environmental, and training-related factors. From a psychosocial perspective, the psychological components, such as mental skills and perceived stress, were shown to influence both performance and well-being. In this sense, a structured mental training program implemented for elite female ice hockey players was shown to significantly reduce burnout and perceived stress while improving decision-making and tactical execution efficiency (Sun et al.). Müller et al. added to these findings by highlighting the importance of organizational structure, personal interactions, and standardized processes for the successful implementation of mental skills development programs. On the other hand, limited financial and human resources were identified as significant barriers, while improved interdisciplinary collaboration (e.g., among teachers, coaches, parents, and sports psychologists) was reported to be a key factor in optimizing resource allocation and facilitating the development of mental skills (Müller et al.).

In the context of training environments, experimental studies showed that different training strategies can effectively enhance specific athletic capacities. Block and undulating periodization both improved sprint performance in young American football players; however, block periodization yielded superior gains (Prioul et al.). Similarly, in a group of 40 male players competing at the college level, the manipulation of task and environmental constraints proved effective in enhancing individual creativity, with combined approaches producing the most substantial improvements (Orangi et al.). Technological advancements were also explored in this special issue, particularly through the application of deep learning techniques to basketball performance analysis (Xiao et al.). This approach enabled the identification of distinct playing styles across leagues and revealed that certain player profiles adapt more consistently to different competitive contexts.

Concurrently, interest in understanding how social environments, including the availability and source of social support, emerged as key determinants of mental health (Luo et al.). The results from this study showed that social support plays a vital role in enhancing athletes’ mental health, particularly through close interpersonal relationships. Specifically, overall social support correlated positively with well-being and negatively with anxiety, depression, and stress. Support from family and friends was found to have a significantly stronger negative association with depressive symptoms than team-based support (Luo et al.). In a similar study, Dongoran et al. emphasized the importance of supportive and developmentally appropriate environments in shaping athletes’ long-term trajectories.

Beyond the social environment, external physical and contextual factors further influence athletes’ experiences and performance. For instance, exposure to air pollution was found to affect the subjective perception of effort in a group of elite female soccer players, while prior exposure to air pollution suggested possible adaptive responses (Beavan et al.). Technological approaches further illustrated how performance is context-dependent, with differences in competitive environments influencing playing styles and adaptation across leagues. Together, these findings highlight that athlete development and performance cannot be understood in isolation from the environments in which athletes are embedded, underscoring the need for integrated, context-sensitive approaches in both research and practice. This body of evidence aligns with the general idea behind the study by Simonti et al. that cultural factors may shape attitudes toward sport psychology services. In this perspective, the authors emphasized the importance of culturally nuanced research to enhance global dialogue and collaboration among sport psychology professionals.

In conclusion, the contributions of this Research Topic provide compelling evidence that athlete development and performance emerge from a complex, dynamic system in which environmental factors are not merely contextual, but central agents. Psychological support structures, training design, technological innovation, and social and cultural contexts interact continuously to shape athletes’ trajectories across all stages of development. These findings reinforce the need to move beyond reductionist perspectives and adopt holistic, interdisciplinary approaches that recognize athletes as embedded within multiple, overlapping environments. Future research should continue to explore these interactions using integrative and ecologically valid methodologies, while practitioners are encouraged to design context-sensitive interventions that account for the specific environmental demands and resources available. Ultimately, advancing our understanding of environmental determinants will be essential to fostering sustainable athlete development, optimizing performance, and promoting well-being in sports.

